# Abundant and active community members respond to diel cycles in hot spring phototrophic mats

**DOI:** 10.1093/ismejo/wraf001

**Published:** 2025-01-08

**Authors:** Amanda N Shelton, Feiqiao B Yu, Arthur R Grossman, Devaki Bhaya

**Affiliations:** Division of Biosphere Sciences and Engineering, Carnegie Science, Stanford, CA 94305, United States; Division of Biosphere Sciences and Engineering, Carnegie Science, Stanford, CA 94305, United States; MultiOmics Tech Center, Arc Institute, Palo Alto, CA 94304, United States; Division of Biosphere Sciences and Engineering, Carnegie Science, Stanford, CA 94305, United States; Division of Biosphere Sciences and Engineering, Carnegie Science, Stanford, CA 94305, United States

**Keywords:** metagenome, metatranscriptome, extremophiles, microbial communities, hot springs, WGCNA, Armatimonadota, pan-genome

## Abstract

Photosynthetic microbial mats in hot springs can provide insights into the diel behaviors of communities in extreme environments. In this habitat, photosynthesis dominates during the day, leading to super-oxic conditions, with a rapid transition to fermentation and anoxia at night. Multiple samples were collected from two springs over several years to generate metagenomic and metatranscriptomic datasets. Metagenome-assembled genomes comprised 71 taxa (in 19 different phyla), of which 12 core taxa were present at high abundance in both springs. The eight most active taxa identified by metatranscriptomics were an oxygenic cyanobacterium (*Synechococcus* sp.), five anoxygenic phototrophs from three different phyla, and two understudied heterotrophs from phylum Armatimonadota. In all eight taxa, a significant fraction of genes exhibited a diel expression pattern, although peak timing varied considerably. The two abundant heterotrophs exhibit starkly different peak timing of expression, which we propose is shaped by their metabolic and genomic potential to use carbon sources that become differentially available during the diel cycle. Network analysis revealed pathway expression patterns that had not previously been linked to diel cycles, including ribosome biogenesis and chaperones. This provides a framework for analyzing metabolically coupled communities and the dominant role of the diel cycle.

## Introduction

The microbial mats in the hot springs of Yellowstone National Park, USA (YNP) have been referred to as the “tropical rainforests” of the microbial world because they harbor dense, diverse, stratified microbial communities dominated by cyanobacteria that use light energy to fix both carbon and nitrogen and other phototrophs [1]. Octopus Spring (OS) and Mushroom Spring (MS) are two geographically close, circumneutral hot springs that have been extensively studied with a focus on the identification of phototrophic organisms, which use (bacterio)chlorophyll to capture light energy for growth, and their abundance as a function of temperature [2]. As water flows away from the spring source, a year-round stable temperature gradient is formed along the outflow channels, and benthic microbial communities with varying compositions are found at different temperatures [3]. Typically, stratified biofilms are formed between 50°C and 70°C, with the top green layer (1–2 mm) dominated by oxygenic cyanobacteria, whereas the orange “undermat” layer harbors mostly anoxygenic phototrophs and less well-characterized heterotrophs [4–6]. Light intensity, quality, and oxygen concentrations vary with mat depth, as does community composition [4, 7–12]. Major events in the diel cycle are as follows: during the day, oxygenic photosynthesis carried out by the unicellular cyanobacterium, *Synechococcus* sp. (recently renamed *Thermostichus* sp.) [13], creates a hyperoxic environment, while carbon fixation causes an increase in the pH of the mat [9, 14]. However, as evening progresses, the rate of aerobic respiration exceeds the rate of photosynthetic O_2_ evolution, and the mats rapidly become anoxic. At night, cyanobacteria switch to fermentation of stored glycogen with an accompanying decrease in pH [7–9]. Filamentous anoxygenic phototrophs such as *Roseiflexus* spp. and *Chloroflexus* spp. abundant in the undermat also fix carbon in the morning, although they use reductants, such as organic acids or hydrogen, produced by other mat organisms [15–17].

Denaturing gradient gel electrophoresis and 16S ribosomal RNA gene amplicon sequencing were used to correlate “ecotypes” of abundant phototrophs with parameters such as temperature, light, and oxygen [3, 12, 18, 19]. This led to the hypothesis that particular ecotypes have evolved to dominate in specific environmental niches [3, 12, 18, 19]. Subsequently, the advent of genomics, metagenomics, and (meta)transcriptomics, coupled with physiological studies, led to the identification of new taxa, revealed complex expression patterns, and high genomic diversity within the mat populations [5, 6, 9, 18, 20–26]. However, gaps remain in our understanding of community interactions, particularly those that are influenced by diel cycles. We reasoned that coupling available biogeochemical data [3, 7, 8, 18, 21, 22, 27–29] with extensive meta-omics analyses could provide insights into the diel behaviors of communities in these extreme environments. In this study, we used samples collected from OS and MS over several years and across a temperature gradient ranging from 50°C to 65°C for metagenomic analyses in conjunction with metatranscriptomic data collected every 2 h over a diel cycle (24 h).

The specific questions we posed were: (i) what are the core and rare taxa, and are they the same in the OS and MS hot springs? (ii) What is the effect of the diel cycle on the expression patterns of the abundant taxa? (iii) Can we use co-expression analysis to discover interlinked behaviors among community members? (iv) What are the roles of abundant taxa in the community based on their patterns of expression?

The key results were that we identified 71 taxa from 19 phyla (species-to-genus-level grouping), of which 12 were considered “core taxa” due to their high abundance in all samples. MS and OS had somewhat different community structures, even though they shared the same abundant and active taxa. We identified eight taxa as most active in the metatranscriptome datasets of which six were phototrophs and two taxa were heterotrophs belonging to the understudied phylum Armatimonadota. A significant fraction of genes in each taxon exhibited a diel expression pattern but with several differences in the peak expression timing between taxa. Despite these significant diel differences, ribosome biogenesis gene expression peaked in the morning in seven of the eight taxa. The two Armatimonadota taxa had differences in gene content and expression timing, suggesting that they have evolved to use different carbon sources released by the phototrophs at different times in the diel cycle. In summary, these analyses provide a more integrated view of the mat community interactions and the role of the diel cycle.

## Materials and methods

### Sample sites and collection

**Figure 1:**
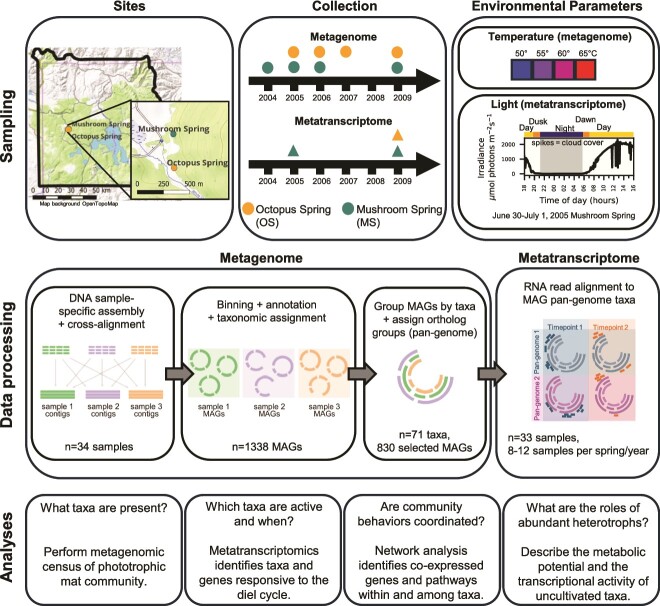
Overview of study site and workflow. Top panels: Sampling. Top left: Mat cores were collected from MS and OS in YNP during the years listed for metagenomics and metatranscriptomics (top middle). Table 1 and Supplementary Data 1 list more specific details. Top right: Environmental parameters for sampling. For light, a typical light curve for MS during metatranscriptome sampling is depicted (adapted from [9]). Sampling at different temperatures was only performed for metagenomes. Middle panels: Data processing: Following DNA extraction, assembly, cross-alignment, and binning, the anvi’o pan-genome pipeline was used to aggregate the selected MAGs into species- to genus-level taxa groups and to filter out MAGs of very low quality or present in only a single sample. Eight hundred thirty MAGs were retained for further analyses, including for the RNA alignment for the metatranscriptome datasets. Bottom panels: Analyses performed to understand the mat community composition and expression.

Mat cores were collected at MS (44.53861 N, 110.79791 W) and OS (44.53401 N, 110.79781 W) on the dates and times provided in Table 1 and Supplementary Data 1, using cork borers (0.5–1 cm) and immediately frozen in liquid nitrogen. Irradiance at the top of the mat was measured on 30 June–1 July 2005, 28–30 July 2009, and 1 October 2005, as described [9] (Fig. 1, Supplementary Data 1). For the MS2005 metatranscriptome, a sample taken at 12:00 p.m. from 1 October 2005 was used to replace the original 1 July 2005 12:00 p.m. sample. Thirty-four mat cores were used for DNA extraction, and 32 were used for RNA extraction.

**Table 1:** Summary of samples.

**TEMP (°C)**	**YEAR**	**SPRING**	**METAGENOME**	**METATRANSCRIPTOME**
60	2004	MS	MS-2004-60-um-a, b	nd
60	2005	MS	MS-2005-60 a, b,c, d, e, f	MS2005 (12 samples over diel cycle)
60	2005	OS	OS-2005-60 a, b	nd
60	2006	MS	MS-2006-60 a, b, c, d, e	nd
60–65	2006	OS	OS-2006-60 a, b, c, d, e, 65a	nd
55	2007	OS	OS-2007-55-um-a, b, c	nd
50–65	2009	MS	MS-2009-60a, 50a, 55a, 60b, 65a	MS2009 (12 samples over diel cycle)
50–65	2009	OS	OS-2009-60a, 50b, 55c, 60d, 65e	OS2009 (8 samples over diel cycle)
**50–65 range**	**5 year range**	**2 springs**	**34 samples (5 years, 4 temperatures)**	**32 TOTAL [2 years, 1 temperature (60)]**

METAGENOME = designations denote spring, year temp and samples taken at different times during the day for metagenome samples, METATRANSCRIPTOME = sampling series name for metatranscriptome samples. Last row (bold): summary of sampling values for each column. Abbreviations: MS, Mushroom Spring; OS, Octopus Spring; letters (a–f) represent individual samples for the metagenome. um, undermat; nd, not done.

### DNA and RNA extraction, library preparation, and sequencing

A modified phenol:chloroform:isoamyl alcohol extraction was performed on mat core samples to extract total DNA and RNA [30]. DNA libraries were created using the KAPA-Illumina library creation kit and sequenced using a 2 × 151 bp indexed run recipe on the NovaSeq S4 (Illumina). For RNA samples, ribosomal RNA (rRNA) was removed with the Ribo-Zero(TM) rRNA Removal Kit (Epicentre) and libraries created with Truseq Stranded RNA LT kit (Illumina) and sequenced the same as the DNA libraries.

### Metagenome assembly, binning, taxonomic identification, and pan-genome analysis

For each metagenome sample, quality control of reads was performed with BBTools v38.26 as described previously [31, 32]. Filtered reads were assembled with SPAdes v3.12.0 [33]. Contigs <200 bp were removed. Genes were called and annotated using the Joint Genome Institute Integrated Microbial Genomes database (JGI/IMG) pipeline (MAP v4.16.5) [34].

Reads from each sample were aligned, using bowtie2 (v2.3.5.1) with default flags, to contigs generated from each sample in order to create a cross-alignment with all possible pairings [35]. Binning, using MetaBAT2, was performed on the contigs using the relative abundance from the cross-aligned dataset [36] to create metagenome-assembled genomes (MAGs). MAGs were taxonomically identified by the best hit in the Genome Taxonomy Database toolkit (GTDB-Tk) (r89) database [37, 38]. MAGs belonging to the same phylum were put into the anvi’o [39] pan-genome pipeline using default parameters. Briefly, the anvi’o pipeline calls genes, uses DIAMOND to compute all similarity scores between all genes, uses the MCL algorithm to identify ortholog groups, and then clusters genomes based on the ortholog groups [39–41]. Pan-genome taxa (genus to species level) were manually selected from each phylum based on the similarity of ortholog groups generated by the pipeline. The taxonomic level with the best match in the GTDB was used as the designation for all MAGs belonging to a pan-genome taxon (Supplementary Data 2). Taxa consisting of single MAGs or only poor-quality MAGs were removed from subsequent analyses. Additional analyses were performed on the anvi’o annotations unless otherwise noted.

The quality of the MAGs was assessed using the Minimum Information about a Metagenome-Assembled Genome (MIMAG) classification [42] (Supplementary Data 2).

### Comparison of pan-genome metagenome-assembled genomes to prior metagenome-assembled genomes

Only about half of the pan-genome taxa were identified to the genus or species level by GTDB-Tk, so to improve resolution of the taxonomic identification, additional curation was performed on select MAGs. We used fastANI (v0.1.3) [43] on Kbase [44] to compare selected MAGs to MAGs generated from the MS undermat [5, 6]. We used the name given by this dataset instead of GTDB-Tk names for MAGs with >94% identity to these selected undermat MAGs [5, 6] (Supplementary Data 2 and 3).

### Metagenome statistical tests

The Shannon Index was calculated using scikit-bio (v0.6) on the relative abundance of the 71 taxa [45] (Supplementary Data 4 and 5). The Kruskal–Wallis test was implemented in scipy (v1.9.3). The Bray–Curtis beta-diversity metric on relative metagenome abundance was calculated using scikit-bio (Supplementary Data 6). Analysis of similarities (ANOSIM) (scikit-bio) and non-metric multidimensional scaling (nMDS) (scikit-learn v1.3.2) of the Bray–Curtis dissimilarity was performed with two dimensions. Analysis of composition of microbiomes (ANCOM) [46] (scikit-bio) with analysis of variance (ANOVA) one-way significance testing was used to evaluate the differential abundance of genera between sample types (Supplementary Data 7).

### Alignment, counting, and normalization of RNA reads to metagenome-assembled genomes

Trimming and filtering were performed with BBTools v38.75 the same as the metagenome reads, with the exception of also using BBMap to remove rRNA reads and known spike-ins [31, 32]. Quality-controlled RNA reads were aligned to 830 MAGs with bowtie2 with default flags [35] (https://doi.org/10.6084/m9.figshare.26530315.v1). Using dereplicated MAGs as a reference for mapping was difficult because some highly abundant and active taxa had fragmented MAGs (Supplementary Data 2) or had closely related genomes that were not easily separated into unique MAGs [5, 18, 25, 26]. To overcome the problem while still capturing a genus- to species-level context for the genes, we mapped reads to all MAGs and summed the counts per ortholog group for each pan-genome taxon. HTSeq-count [47, 48] was used to count read pairs falling within predicted open reading frames (ORFs) with flags (-s reverse -a 0). We set aqual = 0 to account for the expected behavior of reads mapping to multiple locations in the total MAG set due to the presence of conserved regions across MAGs from the same pan-genome. The mapped counts to orthologous gene clusters were summed across all MAGs in the pan-genome taxon (Supplementary Data 8). Gene clusters with low counts across all samples were removed prior to library normalization using edgeR (v3.30.3) using the “RLE” option [49, 50]. A quasi-likelihood negative binomial generalized log-linear model (glmQLfit) fit to a natural spline (spline v 4.0.2) with three degrees of freedom was performed to estimate dispersion independently for each time series. Counts per million (CPMs) were calculated from the normalized counts (Supplementary Data 9). For the convenience of plotting, a single annotation was randomly selected from each gene cluster to use in the analyses (https://doi.org/10.6084/m9.figshare.26530315.v1). We summarized the number of ORFs and ORFs with certain annotations that were detected (at least 1 read) and highly expressed (CPM >1) per pan-genome taxon (Supplementary Data 10).

### Co-expression analysis by consensus weighted gene co-expression network analysis

A signed consensus weighted gene co-expression network analysis (WGCNA) (v1.69) [51, 52] was performed on highly expressed genes (mean CPM >1), with each time series encoded as a separate experiment. Individual co-expression networks were computed using a signed Pearson correlation. Soft-thresholding power (=18) was chosen as recommended in the WGCNA frequently asked questions documentation due to the poor fit of scale-free topology. This was used as input in blockwiseConsensusModules to detect modules and compute eigengenes with the following changes from default: minModuleSize =30, deepSplit = 2, pamRespectsDendro = False, mergeCutHeight = 0.25 (Supplementary Data 11). Eigengenes (Supplementary Data 12) were compared using a Pearson correlation (Supplementary Data 13).

### Overrepresentation analysis

Overrepresentation analysis (ORA) was performed on gene clusters with a Cluster of Orthologous Groups (COG) category [53] using a hypergeometric test [scipy.stats.hypergeom.cdf() (scipy v1.9.3)] and false discovery rate (FDR) multiple testing correction [statsmodels.api.stats.multipletest(method- = “fdr_bh”) v.0.13.5] for each taxon. Adjusted *q*-values <0.05 are reported as significant (Supplementary Data 14).

### Armatimonadota phylogenetic tree

Armatimonadota genomes were downloaded from the National Center for Biotechnology Information (NCBI) Genome database on 13 December 2023. GTDB-Tk de_novo_wf was used to create a concatenated single-copy protein tree of the NCBI genomes, MS undermat MAGs [5, 6], and MAGs from this study and then decorated with Armatimonadota and Chloroflexota genomes from the GTDB-Tk database (R207) [37, 38]. Briefly, following classification by GTDB-Tk, this method calls FastTree with the WAG + GAMMA model on the supplied genomes and then decorates the tree with genomes from the GTDB-Tk database [37, 38, 54]. Tree visualization was performed in the Interactive Tree of Life tool (iTOL) [55].

### Armatimonadota genomic potential

Armatimonadota annotations were analyzed in the following ways. Kyoto Encyclopedia of Genes and Genomes (KEGG) Orthology (KO) numbers from the JGI/IMG annotations and concatenated per taxon for upload to KEGG Reconstruct Pathway for interpretation [56] (accessed 12 October 2022, 22 October 2022). We also examined the annotations created in anvi’o and RASTtk on KBase [44, 57–59]. Some sequences were used as input to the protein Basic Loval Alignment Search (BLASTP) webserver to find similar homologs and annotate NCBI conserved domains from the “Graphic Summary” tab for additional interpretation [60, 61].

JGI-predicted ORFs were used as input to specific pipelines for carbon and energy sources. The GapMind [62] webserver (accessed 13 September 2022–28 September 2022) was used for carbon source prediction. The summary output for each MAG was concatenated for each taxon (Supplementary Data 15). The dbCAN2 web server [63, 64] (accessed 11 October 2022–18 October 2022) was used for carbohydrate-active enzyme (CAZyme) prediction. Genes detected as CAZymes with two or more methods were reported as the total number of genes per CAZy category and normalized by the total number of genes in each MAG (Supplementary Data 16). For iron metabolism and extracellular electron transfer, FeGenie (v1.0) (options --orfs --all_results --heme) [65] was run (Supplementary Data 17 and 18). Genes containing heme-binding domains were run through the NCBI conserved domain database [61] and interproscan [66, 67] to look for domains of interest (accessed September 2023). Putative operon context on assembled contigs was also considered in interpreting function.

### Hydrogenase classification

Sequences annotated as hydrogenases were uploaded to HydDB for classification (accessed 9 March 2023) [68] (Supplementary Data 19).

## Results and Discussion

### Extensive metagenomic sequencing recovers diverse taxa from Mushroom Spring and Octopus Spring

**Figure 2:**
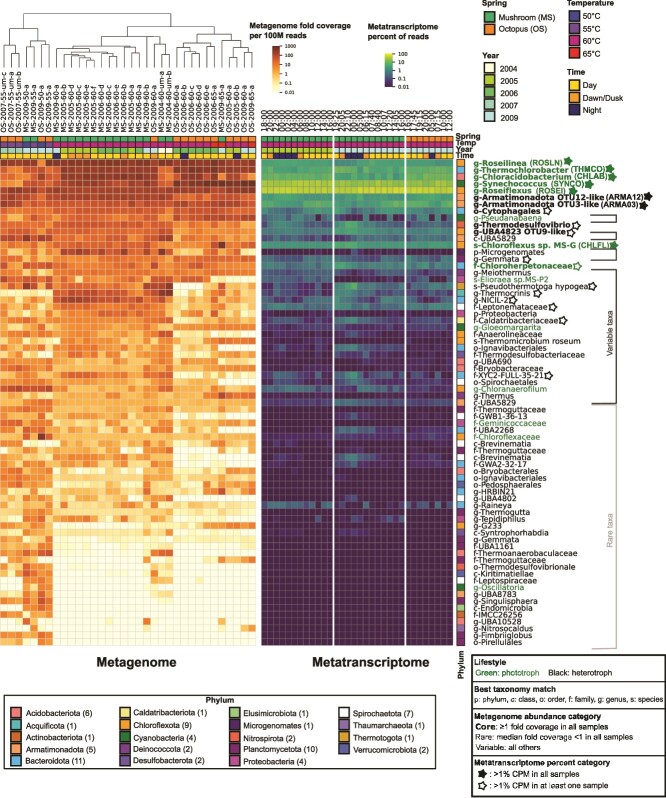
Phototrophic mat microbial community contains core active members, and sample-specific populations. The relative abundance of the 71 genus- to species-level taxa groups obtained from the pan-genome ortholog clustering pipeline was ordered by median relative abundance in all 34 metagenome samples. The metagenome samples are ordered by hierarchical clustering on the relative abundance of the 71 taxa (70 bacterial and 1 archaeal). Metagenome sample names are as in Table 1 and Supplementary Data 1. Sample properties are colored at the top as indicated. Phyla of the 71 taxa are indicated by colored boxes, with the number of taxa identified for each phylum in parentheses after the phylum name. The taxa are identified by the closest relative in GTDB or by best match to other undermat MS MAGs (see Materials and Methods). Abbreviations for best taxonomy match names: p, phylum; c, class; o, order; f, family; g, genus; s, species. Metatranscriptome samples are ordered by sample year and time. Sample properties are colored at the top as indicated. Taxa properties: Green text: Phototrophic taxa based on annotations indicating the presence of photosynthesis reaction center proteins, black text: inferred heterotroph-based lack of reaction center proteins. Bold: Core taxa with at least 1-fold coverage per 100 M reads in all 34 metagenome samples. Rare taxa indicated by light-gray brackets are defined as median coverage of less than one-fold per 100 M reads in the 34 metagenome samples. Variable taxa indicated by dark gray brackets have median coverage between core and rare cutoffs. Filled stars: the eight taxa with over 1% CPM in all 32 metatranscriptome samples; open stars: taxa with at least 1% total counts per million in at least one metatranscriptome sample.

To overcome the limited analytical power typically associated with access to a few samples and/or low sequence coverage, we carried out extensive metagenomic sequencing on the top green layer of mat cores. Most cores were from 60°C sites at either OS or MS collected over 5 years (2004–09). Some samples were taken from four different temperatures in both OS and MS in 2006 and 2009 (Table 1, Figs 1 and 2, Supplementary Data 1). A few samples were also taken from the thick orange undermat (top green layer removed manually) in 2004 and 2007 at 55°C and 60°C (labeled “um” in Table 1, Fig. 2, Supplementary Data 1). These data expand previous sampling efforts; prior published metagenomics performed on MS and OS consisted of four Sanger-sequenced samples across both springs or a single undermat sample [5, 6, 28].

We identified 1338 MAGs (Fig. 1, Supplementary Data 2) (https://doi.org/10.6084/m9.figshare.26530315.v1). One hundred twenty-five MAGs met the MIMAG classification for “High-quality draft MAG”, 535 were “Medium-quality draft,” 466 were “Low-quality draft,” and 212 did not fall into any of the defined categories [42]. To better include abundant but low-quality MAGs in our analysis of community functions, we applied a pan-genome pipeline with manual curation after automated binning and taxonomic identification, see Materials and Methods. The anvi’o pan-genome pipeline [39] was used to identify homologous gene clusters (ortholog groups) between MAGs in the same phylum, and to cluster MAGs into genus- to species-level groups, hereafter referred to as “taxa,” based on the presence of shared ortholog groups in the anvi’o pan-genome viewer (Fig. 1). We filtered out genus- to species-level taxa consisting of a single MAG or very-low-quality MAGs, and 830 MAGs were retained for further analyses. These selected MAGs captured most of the mat phylogenetic diversity as >70% of reads mapped to these (Supplementary Fig. 1).

Of the 830 selected MAGs, 828 MAGs were grouped into 70 taxa from 18 bacterial phyla. The remaining two MAGS were from one taxon in the phylum Thaumarchaeota (genus *Nitrosocaldus*) (Fig. 2, Supplementary Data 2). By comparison, earlier studies from OS and MS phototrophic mats reported only eight bins from four Sanger-sequenced samples, two of which were completely unidentified, whereas 15 bins were recovered from an undermat sample sequenced with Illumina HiSeq [5, 6, 28].

We defined a core set of 12 taxa present in all 34 metagenome samples across both springs and all times and temperatures with at least 1-fold coverage per 100 million reads (Fig. 2, bold names, Supplementary Data 4). These included seven phototrophic taxa, which use (bacterio)chlorophyll to capture light energy for growth. These were from four phyla: Acidobacteriota (*Chloracidobacterium*), Chloroflexota (*Chloroflexus, Roseiflexus*, *Roseilinea),* Cyanobacteriota (*Synechococcus*), and Bacteriodota (*Thermochlorobacter* and one in the family Chloroherpetonaceae). Most of these taxa have been previously observed in hot spring samples from this location [2, 69]. We use *Synechococcus* to describe the most abundant cyanobacterial genus present in this study, although it has been proposed that it be placed in the genus *Thermostichus* because of its evolutionary distance from the type strain [13, 70]. Five heterotrophic taxa were among the core taxa: Armatimonadota OTU3-like, Armatimonadota OTU12-like, a taxon from order Cytophagales from phylum Bacteroidota, *Thermodesulfovibrio* from phylum Nitrospirota*,* and a taxon from the Chloroflexota genus UBA4823 related to OTU 9 from the prior MS undermat metagenome [5, 6]. Of the heterotrophs, only *Thermodesulfovibrio* spp. has a cultured representative from a different hot spring, and the others except for the Cytophagales taxon have been described only in genomic datasets [5, 6, 10, 71]. In this study, we focused on the abundant and active taxa in the community, although we identified 35 “rare taxa” that we defined by a median coverage of <1 from 14 phyla. In addition, there were 24 “variable taxa” from 14 phyla with median coverage values between the core and rare taxa definitions (Fig. 2, Supplementary Data 4). There were four phototrophs in the variable taxa and three in the rare taxa. Some of the variable and rare taxa were present at higher abundances in some samples, and we observed that rare taxa appear to be specifically found at lower temperatures (50°C and 55°C) or in undermat samples. Samples from lower temperatures and the undermat layer had more diverse communities, as indicated by higher Shannon Indices (Supplementary Data 5), which is consistent with previous results [5, 72, 73].

We further explored the community composition of MS and OS and properties that contribute to differences in communities between samples. Both springs are located ~300 m apart in the Lower Geyser Basin of YNP and have similar physical and chemical properties, except the source temperature of OS is 90°C but ~70°C in MS [74, 75] (Fig. 1). Due to the similarity of abundant taxa observed in each spring, these study sites were thought to be very similar in community composition [3, 19, 28]. However, when hierarchical clustering was performed of the relative abundance of the 71 taxa in our data set, OS and MS samples tended to separate (Fig. 2). Analysis of the 60°C sites, for which we had the most samples (*n* = 22), showed that the Shannon Index for alpha diversity was lower in the samples from OS than MS (Supplementary Fig. 2A, Kruskal–Wallis statistic = 9.232, *P* value = .002, Supplementary Data 5). The samples were also significantly different based on the ANOSIM test (*R* = 0.76, *P* value = .001, 999 permutations) on the Bray–Curtis dissimilarity metric for beta diversity (Supplementary Fig. 2B, Supplementary Data 6). Together, these metrics suggest that the taxa composition of the phototrophic mats at 60°C in MS and OS are different. This is consistent with a prior report that the community composition of the overflow water that passes above the phototrophic mats was different between OS and MS, which was attributed to differences in source temperatures [76]. To better understand what was driving this difference, we used the ANCOM method [46], which identified five taxa that were differentially abundant between the two springs. This included two taxa from the phylum Spirochaetota, one from the Bacteroidota, a taxon from phylum Chloroflexota, and a genus from phylum Acquificota (*Thermocrinis*) (Supplementary Fig. 3, Supplementary Data 7). Of these, only *Thermocrinis* sp*.* has been described in some detail, as it was previously isolated from the upper outflow channel of OS [77]. We cannot ascertain whether the differences in community composition are a consequence of chemical, thermal, or biological differences between the springs or a result of historical contingency of colonization. With more intensive sampling of multiple springs [78] or extensive longitudinal sampling, it may be possible to propose hypotheses about specific roles of community interactions, environmental constraints, and evolutionary history on community composition.

### Transcriptional activity measured at 60°C is primarily derived from a few highly active taxa

To identify taxa that contributed most to community transcriptional activity, we carried out metatranscriptomic analysis of samples collected every 2–5 h over a 24 h period at a 60°C site (Fig. 1, Table 1). We used the pan-genomes to aggregate RNA counts per ortholog group in each genus- to species-level taxon generated during the MAG analysis (hereafter referred to as genes), see Materials and Methods. We use the term “expression” as a measure of transcriptional activity, but note that these values do reflect changes in RNA counts as well as possible changes in taxa abundance. We expect that the changes in taxa abundance are relatively minor over the short sampling period (Supplementary Data 6).

Previous metatranscriptomic studies of MS focused on the expression patterns of select genes or taxa of interest over the diel cycle [20–22, 79]. We aimed to analyze the response of the community to the diel cycle by analyzing the expression patterns of the most “active taxa.” We defined “active taxa” as taxa present at the level of at least 1% or higher of the total number of aligned reads in all 32 metatranscriptome samples (varied from 1.2% to 55.6%) (Fig. 2, filled stars). Of these, six taxa were identified as phototrophs [*Roseiflexus* (ROSEI)*, Chloracidobacterium* (CHLAB)*, Thermochlorobacter* (THMCO)*, Roseilinea* (ROSLN)*, Chloroflexus* (CHLFL)*,* and *Synechococcus* (SYNCO)], and two were heterotrophs from phylum Armatimonadota [Armatimonadota OTU3-like (ARMA03) and Armatimonadota OTU12-like (ARMA12)]. For simplicity, we use the abbreviations mentioned in parentheses in some figures. This “first tier” of the most active taxa (Fig. 2, filled stars) is consistent with the most abundant taxa (Fig. 2, bold names). A “second tier” of 11 additional taxa made up at least 1% of total aligned and counted RNA reads at one or more time points (Fig. 2, open stars), but, due to low counts in some samples, their gene expression patterns were not specifically analyzed further.

### Active taxa exhibit peaks of gene expression at different times of the diel cycle

Initially, we sampled some of the highly expressed genes (defined as mean CPM greater than one) in the eight most active taxa to identify diel regulation of genes. Approximately 10% of all genes were highly expressed (24 906 genes out of the 233 893 annotated genes) and were present in 50 of the 71 taxa (Supplementary Data 10). In the eight most active taxa, between 34% and 82% of genes per taxon were highly expressed (Supplementary Data 10).

**Figure 3:**
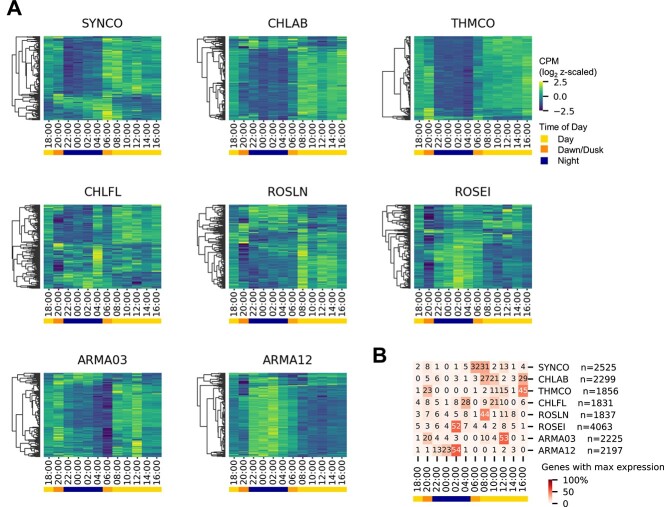
Gene expression of the eight most active taxa varies during the diel cycle. (A) Log2-normalized and *z*-scaled expression in MS2005 of 500 randomly selected highly expressed (mean CPM >1) genes of the eight most active taxa. Sampling of a subset of genes was performed to select genes from across the genome and allow visualization within the limits of the figure. Dendrograms were generated using seaborn clustermap with a correlation distance matrix and complete clustering. (B) Heatmap of percent of highly expressed genes per taxon that had maximum expression at each time point. *n* = number of genes analyzed per taxon.

For an overview of gene expression patterns in each of the active taxa and to determine whether their genes respond to the diel cycle, we visualized the expression patterns of a subset of highly expressed genes (Fig. 3A) and summarized the timing of the maximum expression value (peak) (Fig. 3B, Supplementary Fig. 4). Most gene expression patterns varied as a function of the diel cycle in both the phototrophs and heterotrophs. Six of the eight most active taxa (*Synechococcus*, *Chloracidobacterium*, *Thermochlorobacter*, *Chloroflexus*, *Roseilinea*, and Armatimonadota OTU3-like) had >50% of their genes have their maximum value (peak) during the day (yellow bar Fig. 3B). In contrast, Armatimonadota OTU12-like and *Roseiflexus* had >50% of their genes peaking at night (blue bar Fig. 3B). With the exception of *Roseiflexus*, most phototrophs “turn on” their genes during the day when light energy is available. Closer analysis of expression patterns within the six taxa that had a majority of day-peaking genes revealed a further distinction. Armatimonadota OTU3-like and *Thermochlorobacter* had 20% or more of their genes that exhibited peak expression during dusk. In *Synechococcus,* however*,* 20% or more genes exhibited peak expression at dawn (orange bar Fig. 3B), suggesting particular transcriptional regimes that are more responsive to the transition periods of dusk or dawn. An example of dawn-responsive gene expression previously observed in the mat are genes encoding nitrogenase, which peak at night and a second larger peak at dawn [23] consistent with nitrogenase activity that peaks in the early morning. Of the two Armatimonadota, 53% of Armatimonadota OTU3-like genes had their peak expression at noon (day), whereas, in Armatimonadota OTU12-like, 54% genes had peak expression at 02:00 h (night). This striking contrast in peak expression of the majority of genes suggests that these two taxa may have significantly different metabolic lifestyles, which we explore in detail in the section on Armatimonadota.

### Response to diel cycle in sentinel pathways

**Figure 4:**
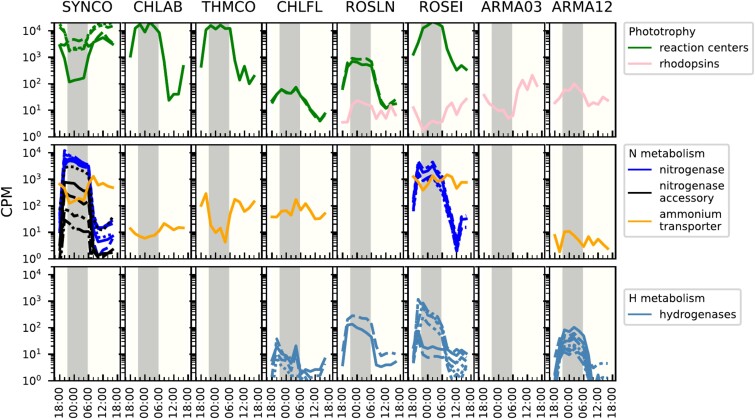
Response of sentinel pathways to the diel cycle in the eight most active taxa. Gene expression from the MS2005 metatranscriptome is plotted for the eight most active taxa. Panels without curves indicate that a gene containing that annotation was not expressed in that taxon. Each line per panel is a separate gene. Gray bar: Night time. CPM, counts per million. Underlying data are found in Supplementary Data 20.

Our analyses suggested that the eight most active taxa could be broadly categorized into either taxa with peak expression during the day or night. To validate and extend this distinction, we examined expression patterns of sentinel genes and pathways known or expected to be regulated by light, anoxia, or circadian rhythms by selecting marker annotations using KO and COG [9, 20–23, 30, 53, 80]. These patterns are visualized for MS2005 (Fig. 4) and MS2009 and OS2009 (Supplementary Fig. 5, Supplementary Data 20).

#### Phototrophy

As expected, *Synechococcus* had higher expression of genes encoding photosynthetic reaction center proteins during the day [9, 20, 23, 81], whereas in the anoxygenic phototrophs, reaction center expression was higher at night [20–22]. In addition, we observed rhodopsin expression in four taxa that might be indicative of an alternative to (bacterio)chlorophyll-based phototrophy (Figs 2 and 4, names in green). Rhodopsin expression was higher at night in *Roseilinea* but higher during the day in *Roseiflexus* (Fig. 4, Supplementary Fig. 5). Both Armatimonadota rhodopsins were not specifically responsive to the diel cycle. Canonically, rhodopsin expression is regulated by light [82], but our results suggest that there are additional inputs into the regulation of rhodopsins in these taxa, for example, by nutrient levels as described in *Vibrio* spp. [83].

#### Nitrogen fixation and assimilation

Nitrogenase activity in the unicellular *Synechococcus* has been shown to have peak activity in the early morning consistent with the model that nitrogenase requires high levels of adenosine triphosphate (ATP) and reductant, which are readily available in the early morning hours when photosynthesis is increasing. Our metatranscriptomic data confirm and extend these results by showing that *nifHDK* as well as nitrogenase accessory subunit gene expression patterns follow the previously observed trend of a small peak at night and a second spike in the morning (Fig. 4, Supplementary Fig. 5) [23, 30]. Nitrogenase accessory genes were not encoded or expressed in *Roseiflexus* MAGs, consistent with previous genome analyses and a report that the assembly of the *Roseiflexus* core proteins can occur without these accessory proteins [6, 84, 85]. We observed the expression of ammonium transporters (Amt) in six of the eight taxa, which suggests these use ammonia as their nitrogen source.

#### Hydrogenases

H_2_ concentration increases in the mats during the night with a second peak at dawn, possibly concurrent with nitrogenase activity, since hydrogen is a byproduct of nitrogenase [7, 8]. Consistent with this, hydrogenases in Armatimonadota OTU12-like, *Chloroflexus*, and *Roseiflexus* exhibited elevated levels of expression at night when the mats are anoxic and amenable to enzyme assembly (Fig. 4, Supplementary Fig. 5). *Synechococcus* appears to lack hydrogenases, suggesting that the hydrogen it produces by fermentation and nitrogen fixation activity could serve as a source of reductant/energy for hydrogenase-containing organisms in the mat.

### Consensus module detection reveals co-expression of genes within and between taxa

**Figure 5:**
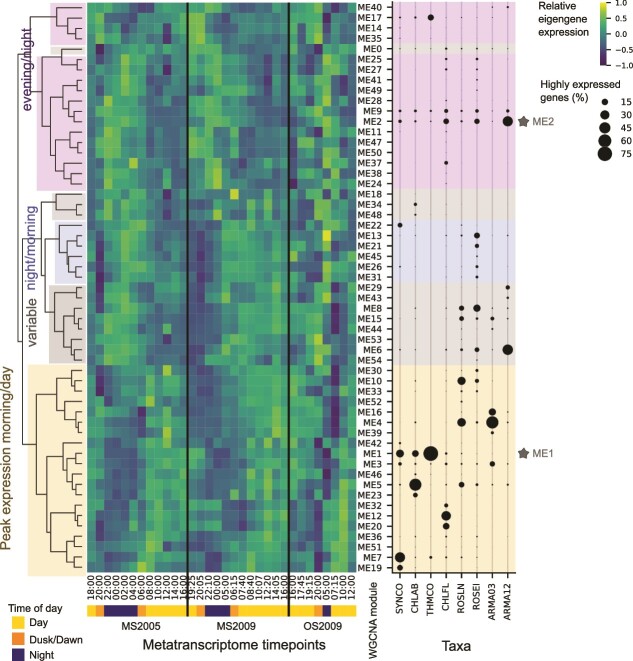
Consensus WGCNA modules in show differences and similarities in expression patterns between taxa. Highly expressed genes from all taxa were separated into modules through consensus-module WGCNA analysis, and each module is represented by its eigengene. To find the relationship between the modules, eigengene expression patterns (Supplementary Data 12) for the 54 modules and ME0 across all three sampling series were hierarchically clustered using the Pearson correlation (Supplementary Data 13). Labels on the dendrogram describe the relationship between eigengenes. Except for the variable category, eigengenes had qualitatively similar peak expression times in each of the three time series (MS2005, MS2009, and OS2009). The fraction of the highly expressed genes in each WGCNA module of the eight active taxa shows the distribution of genes across WGCNA modules. The two largest WGCNA modules (ME1 and ME2) are starred in gray.

To discover biological activities or interlinked behaviors between microbes in the community, we identified groups of genes with similar expression patterns across the entire community using WGCNA with consensus module detection on the highly expressed genes of the metatranscriptomes [51, 52]. Most of the highly expressed genes (24 701 of 24 906 genes) were placed into 54 clusters of genes that are co-expressed in all three time series (called modules) (ME1–54) (Fig. 5). Only a few remaining genes (205, labeled ME0) could not be placed in a specific module (Supplementary Data 11). The eigengene, the first principal component, was used to summarize the expression pattern of each module (Supplementary Data 12).

#### Genes in the same weighted gene *c*o-expression network analysis module show co-expression

By performing simultaneous WGCNA on all organisms, we were able to infer if there was co-expression between taxa (i.e. genes in the same WGCNA module indicate high correlation). We found that most WGCNA modules tend to be dominated by a single taxon. In 16 of the 54 modules, 90% of the genes were from a single taxon, and >50% of the genes in 47 of the 54 modules were from a single taxon (Supplementary Fig. 6). Thus, we hypothesize that gene expression is not very strongly coordinated “between” taxa but primarily “within” taxa or, in other words, it is “organism specific.” However, we did find examples where six modules contained genes from >20 taxa (Supplementary Fig. 6). This included the largest WGCNA modules, ME1, which is a morning/day-peaking module (*n* = 2682 genes, 21 taxa), and ME2, which is an evening/night-peaking module (*n* = 2521 genes, 40 taxa) (Fig. 5). This result suggests that there is coordination or convergence in expression patterns across taxa.

#### Relationships between weighted gene *c*o-expression network analysis modules show similarities in expression timing

We observed limited co-expression in many modules based on the inclusion of multiple genera in each module, but the method is sensitive to the magnitude of gene expression changes which varies widely across taxa. To see if similarities could be inferred by expression timing, modules were hierarchically clustered and categorized by peak expression timing (Fig. 5, Supplementary Data 13). Four taxa had between 38% and 78% of their highly expressed genes in a single module in the morning/day-peaking category. This included Armatimondota OTU3-like, *Chloracidobacterium*, *Thermochlorobacter,* and *Synechococcus* (Fig. 5). This leads us to postulate that in these taxa a predominant mode of behavior is driven directly or indirectly by the diel cycle. A striking exception to this behavior was shown by the Armatimonadota OTU12-like taxon, which had 37% of its highly expressed genes in an evening/night module (ME2). A third pattern of gene expression exhibited by the three Chloroflexota taxa was more complex, and module sizes were generally smaller in these than the other taxa. In *Chloroflexus* and *Roseilinea* the largest modules were morning/day-peaking, whereas those in *Roseiflexus* were night-peaking or had no consistent expression patterns across all three time series (Fig. 5).

### Overrepresentation of biological functions in weighted gene co-expression network analysis modules reveals similarities in expression timing

**Figure 6:**
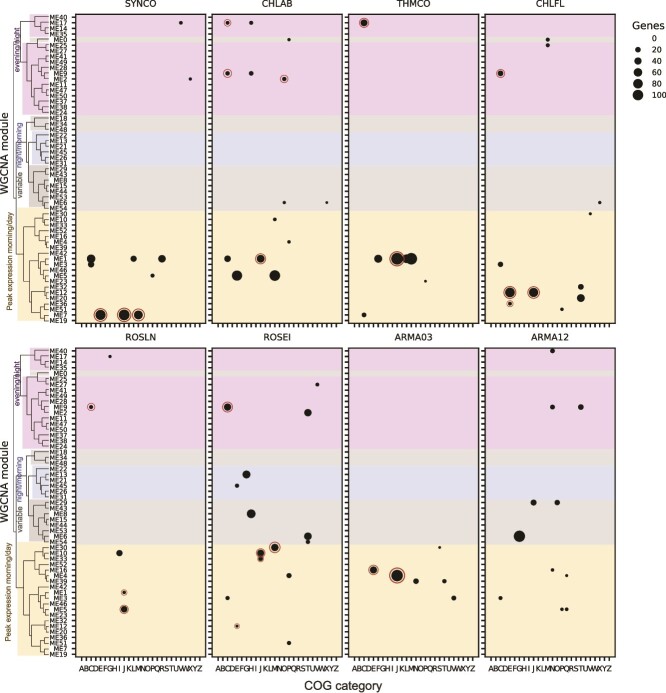
COG category overrepresentation per WGCNA module in active taxa finds timing of biological functions. Significantly overrepresented COG categories per WGCNA module are shown for the eight active taxa. Circle size represents the number of genes. Module ordering by the dendrogram and coloring is the same as in Fig. 5 based on the hierarchical clustering of module eigengenes and peak expression time. Associated data are found in Supplementary Data 14. Modules discussed in the text are circled in dark red. COG category definitions: (A) RNA processing and modification; (B) chromatin structure and dynamics; (C) energy production and conversion; (D) cell cycle, division, chromosome partitioning; (E) amino acid transport and metabolism; (F) nucleotide transport and metabolism; (G) carbohydrate transport and metabolism; (H) coenzyme transport and metabolism; (I) lipid transport and metabolism; (J) translation, ribosome structure and biogenesis; (K) transcription; (L) replication, recombination and repair; (M) cell wall/membrane/envelope biogenesis; (N) cell motility; (O) post-translational modification, protein turnover, chaperones; (P) inorganic ion transport and metabolism; (Q) secondary metabolite biosynthesis, transport and catabolism; (R) general function prediction only; (S) function unknown; (T) signal transduction mechanisms; (U) intracellular trafficking, secretion, and vesicles; (V) defense mechanisms; (W) extracellular structures; (X) mobilome: prophages, transposons; (Y) nuclear structure; (Z) cytoskeleton.

To delve into the different patterns of gene expression revealed by the WGCNA modules, we asked if specific biological functions, defined by COG categories, were overrepresented in the modules (see Materials and Methods for details on ORA) [53]. We found 89 examples of COG categories in the active taxa that were significantly overrepresented in modules (Fig. 6, Supplementary Data 14).

We examined three COG categories where there was conservation in the timing of biological functions between taxa.

#### Translation and ribosome structure and biogenesis (J category)

**Figure 7:**
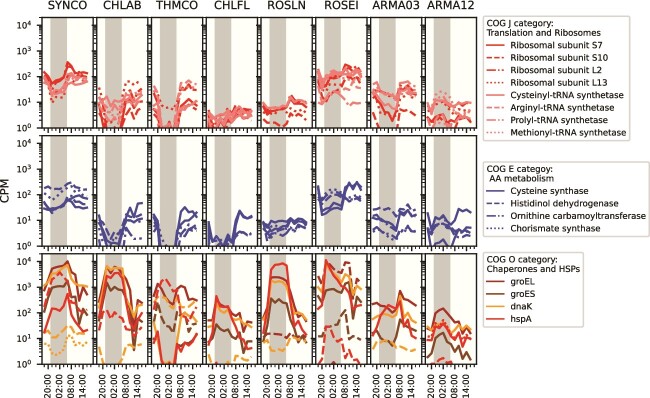
Expression patterns of selected genes from the COG category overrepresentation analysis. A subset of genes from COG categories of interest were selected to be visualized for the eight most active taxa using the MS2005 metatranscriptome. Each line is a different gene. Top: J category, translation and ribosomes. Middle: E category, amino acid (AA) metabolism. Bottom: O category, chaperones and heat shock proteins (HSPs). CPM, counts per million. Underlying data are found in Supplementary Data 21.

This group was significantly overrepresented in morning/day-peaking modules in all six phototrophs and Armatimonadota OTU3-like (Fig. 6, circled data). Selected example genes are plotted (Fig. 7, Supplementary Fig. 7, Supplementary Data 21). We infer that these seven taxa have high rates of protein synthesis in the morning and day. We hypothesize that as photosynthesis peaks during the day, it provides the energy required for energetically intensive processes such as protein synthesis and ribosome biogenesis. This timing is similar to what has been observed in marine metatranscriptomes, where ribosome transcripts with a 24-h periodicity peak in the morning or shortly before dawn in both cyanobacteria and heterotrophs [82, 86]. The exception is *Roseiflexus,* where many genes are highly expressed at night. In this case, we hypothesize that transcription is not as strongly coupled to translation, as in other taxa. To test such a hypothesis, coupled transcriptional and proteomic and functional data are required, but only a few such studies are available [9, 23]. A metatranscriptomics study of MS observed a temporal discrepancy in *Roseiflexus* between transcriptional profiles and light-driven CO_2_ fixation and suggested it may be due to post-translational regulation [22]. Finally, an exception to this pattern of the high expression during the morning or day is seen in Armatimonadota OTU12-like, where ribosome expression is highest at night, which is consistent with the finding that most of its genes have peak expression at night (Figs 3 and 5). In summary, ribosome peak expression does appear to track with the peak gene expression in each taxon.

#### Amino acid biosynthesis and cell wall biosynthesis (E and M categories)

Categories related to cell growth were significantly overrepresented in morning or day-peaking modules in some taxa (Fig. 6, circled data). In Armatimonadota OTU3-like, *Chloroflexus, Roseiflexus,* and *Synechococcus*, amino acid biosynthesis (E category) was significantly overrepresented (Fig. 7, Supplementary Fig. 7, Supplementary Data 21). This is consistent with *in situ* metabolomics showing that peak amino acid biosynthesis in the mat occurs in the late morning [29]. Cell wall biosynthesis genes (M category) were significantly overrepresented in *Roseiflexus* (ME30) and *Synechococcus* (ME7), also suggesting that these two taxa have similar timings of cell growth and division (Fig. 6, circled data). Therefore, despite differences in pigmentation and sources of fixed carbon and reductant [2], many of the phototrophs have increases in the expression of genes associated with cell growth and division in the morning, suggesting some convergence in the timing of the cell cycle as sunlight becomes available for photosynthesis. We speculate that Armatimonadota OTU3-like may get the energy needed for these functions by consuming the photosynthate produced by the phototrophs. Additional *in situ* data types such as quantification of dividing cells, protein synthesis rates, or laboratory experiments monitoring the cell cycle will help confirm that the high expression of these genes is correlated with an increase in cell growth and division in the morning.

We also used the ORA approach to discover pathways that are active in the absence of light energy in the phototrophs.

#### Energy production and conversion (C category)

Some C category genes of the anoxygenic phototrophs *Thermochlorobacter* (ME17), three Chloroflexota (ME9) and *Chloracidobacterium* (ME9 and ME17) were significantly overrepresented in night-peaking modules (Fig. 6, circled data). These genes encoded hydrogenases (Fig. 4), and proteins expected to be more highly expressed at night, including quinol oxidases, NADH oxidoreductases, ferredoxins, and flavodoxins.

#### Post-translational modification, chaperones, and heat shock proteins (O category)

Genes from this category were significantly overrepresented in a night-peaking module (ME2) in *Chloracidobacterium* (Fig. 6, circled data). The occurrence of a night-specific chaperone response prompted us to determine if this was unique to *Chloracidobacterium* or a more general phenomenon. We examined expression patterns of genes encoding specific chaperones across the other seven active taxa (Fig. 7, Supplementary Fig. 7, Supplementary Data 21). The four other anoxygenic phototrophs exhibited a relative increase in expression of some of these chaperones at night. In contrast, *Synechococcus* chaperones tended to peak around dawn. Some taxa had multiple *dnaK* orthologs exhibiting opposite expression patterns, suggesting different roles for each copy (Fig. 7, Supplementary Fig. 7, Supplementary Data 21). We hypothesize that the timing of expression may be synchronized to peak at specific times for reasons such as (i) controlling processes that are particularly susceptible to light or oxygen, (ii) a link to a specific phase of the cell cycle, as in *Caulobacter crescentus,* and (iii) being controlled by the cyanobacterial circadian clock [87, 88].

#### Abundant Armatimonadota have different predicted carbon sources and timing of expression

**Figure 8:**
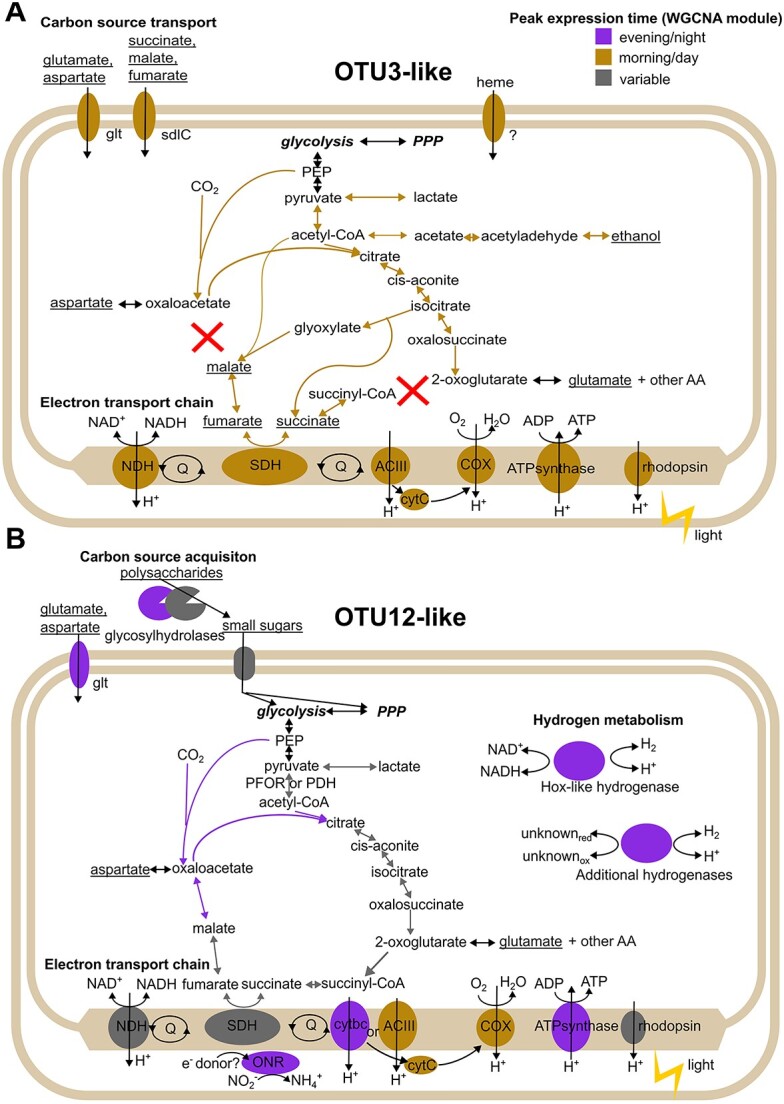
Expression times of major metabolic genes in Armatimonadota OTU3-like and OTU12-like vary between taxa (A) Armatimonadota OTU3-like, (B) Armatimonadota OTU12-like. Colors of enzymes are based on their WGCNA module and eigengene expression time (Fig. 5). Underlined metabolites were predicted to be used by the GapMind webserver. Abbreviations: PPP, pentose phosphate pathway; PEP, phosphoenolpyruvate; AA, amino acids; NDH, NADH dehydrogenase; SDH, succinate dehydrogenase; Q, quinone pool; ACIII, alternative complex III; COX, cytochrome c oxidase; cytC, cytochrome c; cytbc, cytochrome bc; ONR, putative octoheme nitrite reductase; glt, glutamate transporter; sdlC, dicarboxylate transporter.

Several of the active and abundant phototrophic taxa have been described [15, 17, 21, 22, 24, 81], whereas the role of heterotrophs in the alkaline hot spring phototrophic mats is underexplored [10, 79]. We detected MAGs from five genera from three classes in the phylum Armatimonadota, present in both springs at multiple temperatures (Supplementary Fig. 8, Supplementary Data 2). Armatimonadota OTU3-like from class Fimbirimonadia and Armatimonadota OTU12-like from class HRBIN16 were among the most active genera at 60°C and have been previously reported in MS undermat metagenome samples [5, 6] (Fig. 2). We hypothesized that their starkly different expression patterns [either day-peaking (Armatimonadota OTU3-like) or night-peaking (Armatimonadota OTU12-like)] may be linked to their metabolic potential, so we carried out additional manual curation on their MAGs with a focus on carbon and energy utilization pathways (see Materials and Methods) (Fig. 8, Supplementary Data 15–19).

Armatimonadota OTU3-like has the capacity to use fatty acids and certain amino acids as carbon sources (Fig. 8A, Supplementary Data 15). Like in *Fimbriimonas ginsengisoli* [89], a better-characterized member of this class, there is an incomplete tricarboxylic acid (TCA) pathway lacking malate dehydrogenase and oxoglutarate dehydrogenase, but it has glyoxylate shunt enzymes malate synthase and isocitrate dehydrogenase, suggesting an alternative flux compared to the canonical TCA cycle. Although Armatimonadota OTU3-like has an aerobic respiratory pathway with alternative complex III, it lacks heme biosynthesis, suggesting that it must acquire it from the environment. Consistent with this, subunits of candidate heme transporters were identified and expressed *in situ* (Supplementary Data 18). We did not detect any pathway for anaerobic respiration, but it may be able to ferment via the activity of a lactate, an aldehyde, or an alcohol dehydrogenase. We observed the expression of a rhodopsin gene (Fig. 4), suggesting that Armatimonadota OTU3-like may be able to supplement its energy production using light (Fig. 8A). Many of these pathways are upregulated during the day (Figs 3–6 and 8A), suggesting that its preferred carbon sources are more bioavailable during the day. Consistent with this observation, whole mat polar metabolomics suggested that an increase in the relative abundance of some organic acids that Armatimonadota OTU3-like is predicted to use in the afternoon [29].

In contrast, Armatimonadota OTU12-like is predicted to use small sugars including sucrose, cellobiose, glucose, and xylose (Fig. 8B, Supplementary Data 15). Carbohydrate metabolism (COG G category) was overrepresented in ME6, highly expressed in the evening, night, or early morning in the three metatranscriptome series (Figs 5 and 6). More secreted glycosylhydrolases per MAG were predicted by dbCAN2 in Armatimonadota OTU12-like than Armatimonadota OTU3-like (mean = 16.5 vs 5 per MAG), suggesting that Armatimonadota OTU12-like can degrade extracellular polysaccharides (Supplementary Data 16). Whether these carbohydrates are found in the extracellular polymeric substances (EPS) of the mat is not yet known. Unlike Armatimonadota OTU3-like, Armatimonadota OTU12-like contains a complete TCA cycle, multiple bidirectional hydrogenases, a candidate octoheme nitrite reductase, and multiheme cytochromes of unknown function that are predicted to be membrane-embedded (Supplementary Data 17–19). This suggests that there may be alternative electron flow pathways that switch between anaerobic respiration at night and aerobic respiration in the day (Fig. 8B). Like Armatmonadota OTU3-like, Armatimonadota OTU12-like also harbors an expressed rhodopsin gene (Figs 4 and 8B). A lactate dehydrogenase was also identified, suggesting the potential for fermentation. Many genes are upregulated at night (Figs 3–6 and 8B), although how timing of gene expression relates to the local availability of saccharides is not known. These results suggest differential diel activities and metabolisms of the Armatimondota in hot spring phototrophic mats, which was only previously inferred by genome sequences [5, 6, 90]. These data also suggest possible enrichment strategies to obtain these organisms for which no type strain is currently available.

Here we present a detailed census of microbial taxa present in OS and MS and identified twelve taxa as the core members of the MS and OS mat community. However, relative abundances suggest differences in community compositions between each spring and further support the conclusion that somewhat different communities exist at different temperatures. We identified eight taxa which are the most transcriptionally active members of the community at 60°C and all had gene expression patterns that correlated with the diel cycle. This revealed possible interactions between taxa. Our analysis not only revealed some similarities in expression patterns for pathways and functions but also highlights major differences in expression patterns such as in the two Armatimonadota.

This sets the stage for understanding community level interactions but we acknowledge some caveats about our datasets and current conclusions. For instance, we do not know to what extent diel variation in transcript abundances is reflected at the protein level [30, 91, 92]. Temporal shotgun metaproteomics of the mat will help determine this; so far, such analyses have only been performed for single time points [16, 93]. We know that EPSs are important for mat structure and may be a source of organic carbon for some mat organisms, as has been observed in other types of microbial mats [94–96]. Although whole mat shotgun polar small molecule metabolomics has been performed [29], the dynamics of EPS composition and abundance have not been characterized. We have evidence of metabolite transfer between cyanobacteria and filamentous cells from previous radio-labeling experiments [97, 98]; however updated techniques will be better able to resolve the identity of both the organisms and metabolites involved. These include activity measurements *in situ*, such as stable isotope probing (SIP)-metagenomics with different labeled carbon isotopes [99] or SIP-metaproteomics [100]. A fine-grained understanding of spatiotemporal mat dynamics would address community abundance, dynamics, and activity as a function of mat depth and diel fluctuation in light or oxygen levels.

Whether some of the observations from this study are a common feature of microbial communities and whether the diel cycle is a major driver of community interactions remain open questions. One ecosystem where the effect of the diel cycle on community transcriptional activity has been extensively studied is the open ocean, but it is very different from a hot spring phototrophic mat in nutrient composition, spatial structure, and cell density. Like mat systems, much of the analysis has focused on the responses of abundant phototrophs, which have similar timing for major metabolic pathways across habitats. However, some studies have also observed diel responsive genes (i.e. with a 24-h periodicity) in some heterotrophs [82, 86]. Differential timing of uptake transporters in marine microbial community members supports the concept of diel niche partitioning in this system as well [86]. Future work may allow us to connect the observations of diel community activity in mat and ocean systems to other microbial communities, including systems that are less dominated by phototrophs where the diel cycle may play an underappreciated role.

## Supplementary Material

SupplementaryData1_wraf001

SupplementaryData2_wraf001

SupplementaryData3_wraf001

SupplementaryData4_wraf001

SupplementaryData5_wraf001

SupplementaryData6_wraf001

SupplementaryData7_wraf001

SupplementaryData8_wraf001

SupplementaryData9_wraf001

SupplementaryData10_wraf001

SupplementaryData11_wraf001

SupplementaryData12_wraf001

SupplementaryData13_wraf001

SupplementaryData14_wraf001

SupplementaryData15_wraf001

SupplementaryData16_wraf001

SupplementaryData17_wraf001

SupplementaryData18_wraf001

SupplementaryData19_wraf001

SupplementaryData20_wraf001

SupplementaryData21_wraf001

RevisedSupplementaryInformationv2_wraf001

## Data Availability

Datasets generated and analyzed in the current study are available in the SRA, NCBI, JGI/IMG, and figshare repositories as detailed below or included as supplemental data in this published article. Raw sequence reads can be found on the SRA at SRP191317, SRP191318, SRP191319, SRP191321, SRP191322, SRP191324, SRP191332, SRP191328, SRP191329, SRP191333, SRP213393, SRP213396, SRP213395, SRP213397, SRP213394, SRP191337, SRP213390, SRP213392, SRP213391, SRP191334, SRP213388, SRP191350, SRP213398, SRP213402, SRP239941, SRP213411, SRP213409, SRP213410, SRP213412, SRP213413, SRP213403, SRP213408, SRP213407, SRP213406, SRP259901, SRP259948, SRP259967, SRP260043, SRP259966, SRP260069, SRP260079, SRP260124, SRP260130, SRP260133, SRP260135, SRP260129, SRP288870, SRP288866, SRP288863, SRP288864, SRP288862, SRP288861, SRP288868, SRP288867, SRP288819, SRP288812, SRP288826, SRP288804, SRP288815, SRP288817, SRP288820, SRP288824, SRP288827, SRP288823, SRP288853, SRP299000. NCBI Bioprojects associated with the samples are: PRJNA518216, PRJNA518217, PRJNA518218, PRJNA518219, PRJNA518220, PRJNA518221, PRJNA518222, PRJNA518223, PRJNA518224, PRJNA518225, PRJNA539609, PRJNA539610, PRJNA539611, PRJNA539612, PRJNA539608, PRJNA518227, PRJNA539605, PRJNA539606, PRJNA539607, PRJNA518226, PRJNA539604, PRJNA518228, PRJNA539613, PRJNA539614, PRJNA571079, PRJNA539619, PRJNA539620, PRJNA539621, PRJNA539622, PRJNA539623, PRJNA539615, PRJNA539618, PRJNA539617, PRJNA539616, PRJNA621571, PRJNA621572, PRJNA622232, PRJNA621573, PRJNA621574, PRJNA621575, PRJNA621576, PRJNA621577, PRJNA621578, PRJNA621580, PRJNA621581, PRJNA621579, PRJNA653764, PRJNA653765, PRJNA653766, PRJNA653767, PRJNA653768, PRJNA653769, PRJNA653770, PRJNA653771, PRJNA653752, PRJNA653753, PRJNA653754, PRJNA653755, PRJNA653756, PRJNA653757, PRJNA653758, PRJNA653759, PRJNA653760, PRJNA653761, PRJNA653762, PRJNA677357. JGI/IMG portal accessions can be found in Supplementary Data 1. MAG sequences and annotations, and some additional data used in plotting have been deposited at Figshare: https://doi.org/10.6084/m9.figshare.26530315.v1 Additional analyzed datasets are provided as Supplementary Data 1–21. Descriptions of these Supplementary Data are found in Supplementary Information.

## Code availability

Custom scripts used to generate and analyze data can be found in our repository on GitHub: https://github.com/carnegie/YNP_MSOS_metagenomics_metatranscriptomics
