# Supplementary material for: Abundant and active community members respond to diel cycles in hot spring phototrophic mats
Source: ISME J. 2025 Jan 8;19(1):wraf001. doi: 10.1093/ismejo/wraf001 (PMC11788075; doi:10.1093/ismejo/wraf001)
Supplement: RevisedSupplementaryInformationv2_wraf001 [file revisedsupplementaryinformationv2_wraf001.pdf]

## Supplementary Materials for

# Abundant and active community members respond to diel cycles in hot spring phototrophic mats

Amanda N. Shelton[1*], Feiqiao B. Yu[1,2], Arthur R. Grossman[1], Devaki Bhaya[1*]

1. Division of Biosphere Sciences and Engineering, Carnegie Science, Stanford, CA, USA
2. Arc Institute, Palo Alto, CA

*Correspondence should be directed to Amanda N. Shelton (ashelton@carnegiescience.edu) or Devaki Bhaya (dbhaya@carnegiescience.edu)

**This file contains:**
Supplementary Figures 1-8
Description of Supplementary Data 1-21
**Other Supplementary Material includes the following:**
Supplementary Data 1-21

## Description of Supplementary Data Files

**Supplementary Data 1: Sample accessions and metadata**

**Supplementary Data 2: MAG metadata**
Samples metadata draws from **Supplementary Data 1**. MAG metadata includes genome size, number of contigs, GC content, estimates of completeness and contamination from checkM, the fold coverage of the MAG in the sample it is from, number of annotations from JGI/IMG and anvi'o pipelines, whether the MAG was selected for further analysis, the pan-genome lineage designation and the full GTDBtk results per MAG. MIMAG classification is also included.

**Supplementary Data 3: fastANI results comparing selected MAGs**

**Supplementary Data 4: Relative abundance of pan-genome taxa in metagenome samples**

**Supplementary Data 5: Metagenome Shannon indices**

**Supplementary Data 6: Bray-Curtis dissimilarity matrix for metagenome samples**

**Supplementary Data 7: ANCOM results for spring-specific taxa in 60°C metagenomes**
The percentile columns show the relative metagenome coverage for that taxon in each spring at the different percentile values for the taxon. The W-statistic is the number of taxa that the taxon has been tested to be significantly different against.

**Supplementary Data 8: RNA mapping output from HTSeq-count aggregated per ortholog group in each pan-genome taxon group**

**Supplementary Data 9: Normalized RNA counts per million per times series using the pan-genome taxon ortholog groups**
Each sheet is a different sampling series

**Supplementary Data 10: Summary statistics on metatranscriptome counts per gene and annotation type**
The total ORFs includes hypothetical genes, "annotated" is only genes with a KOfam or COG annotation. For "detected" columns, this represents at least one read per gene, and "highexpress" represents a mean CPM > 1 in that time series.

**Supplementary Data 11: WGCNA module assignment to genes**

**Supplementary Data 12: Eigengene expression values**

**Supplementary Data 13: Pearson Correlation of WGCNA module eigengene expression patterns**

**Supplementary Data 14: Overrepresentation analysis results for COG Categories in WGCNA modules in pan-genome taxa.**

**Supplementary Data 15: GapMind carbon source prediction results on Armatimonadota OTU3-like and Armatimonadota OTU12-like.**

**Supplementary Data 16: dbCAN carbohydrate active enzymes prediction results on Armatimonadota OTU3-like and OTU12-like**

**Supplementary Data 17: FeGenie heme binding protein prediction results on Armatimonadota OTU3-like and Armatimonadota OTU12-like.**

**Supplementary Data 18: FeGenie results for other categories on Armatimonadota OTU3-like and Armatimonadota OTU12-like.**

**Supplementary Data 19: HydDB classification of genes annotated as "hydrogenase" in the eight most active taxa.**
Only the catalytic subunit is classified by the database into the different categories, other subunits give a "NONHYDROGENASE" result.

**Supplementary Data 20: Selected expression data of sentinel genes for Figure 4 and associated Supplementary Figure 5.**
The annotation is given for both COG and KOfam accessions. Sample names correspond to those in **Supplementary Data 1.**

**Supplementary Data 21: Selected expression data for genes for Figure 7 and Supplementary Figure 7.**
The annotation is given for both COG and KOfam accessions. Sample names correspond to those in **Supplementary Data 1.**

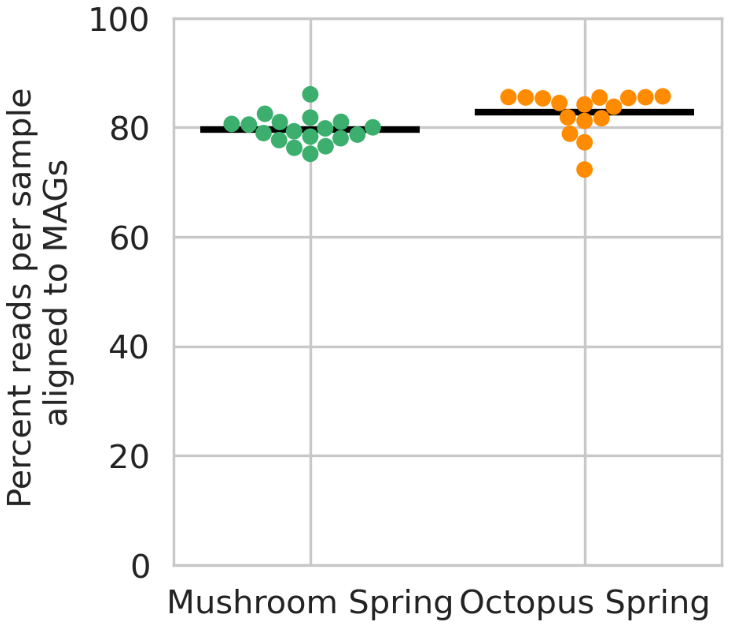


**Supplementary Figure 1: Percent of reads mapping to MAGs**
Alignment of metagenome paired reads to the 830 selected MAGs for all 34 metagenome samples. Mean percent reads aligned per spring are indicated with the black line.

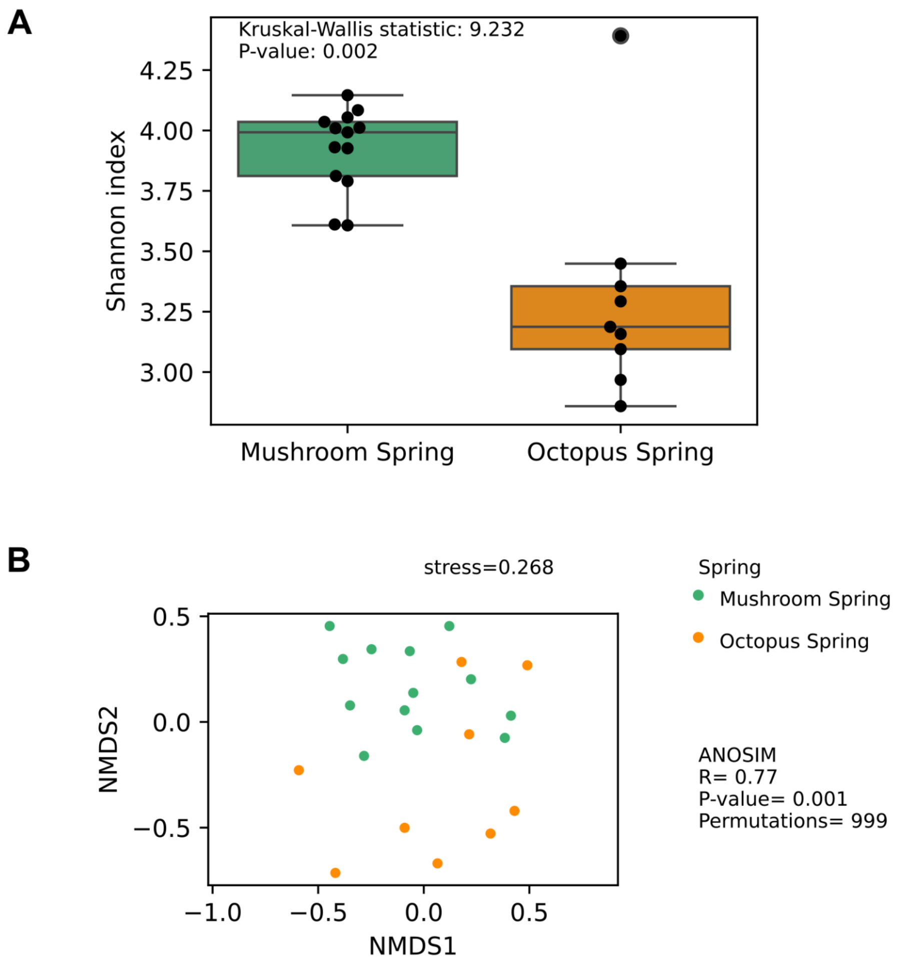


**Supplementary Figure 2: Diversity metrics of 60°C samples**
**(A). Shannon alpha diversity index on metagenome relative abundance.** Boxplots show the median, upper and lower quartiles of the Shannon index. Whiskers are 1.5 times the interquartile range, and points are those falling outside the whiskers. **(B). Bray-Curtis dissimilarity of the metagenome relative abundance.** 2-D nMDS on Bray-Curtis dissimilarity is plotted. The ANOSIM test was performed to compare Mushroom Spring and Octopus Spring, total samples =22.

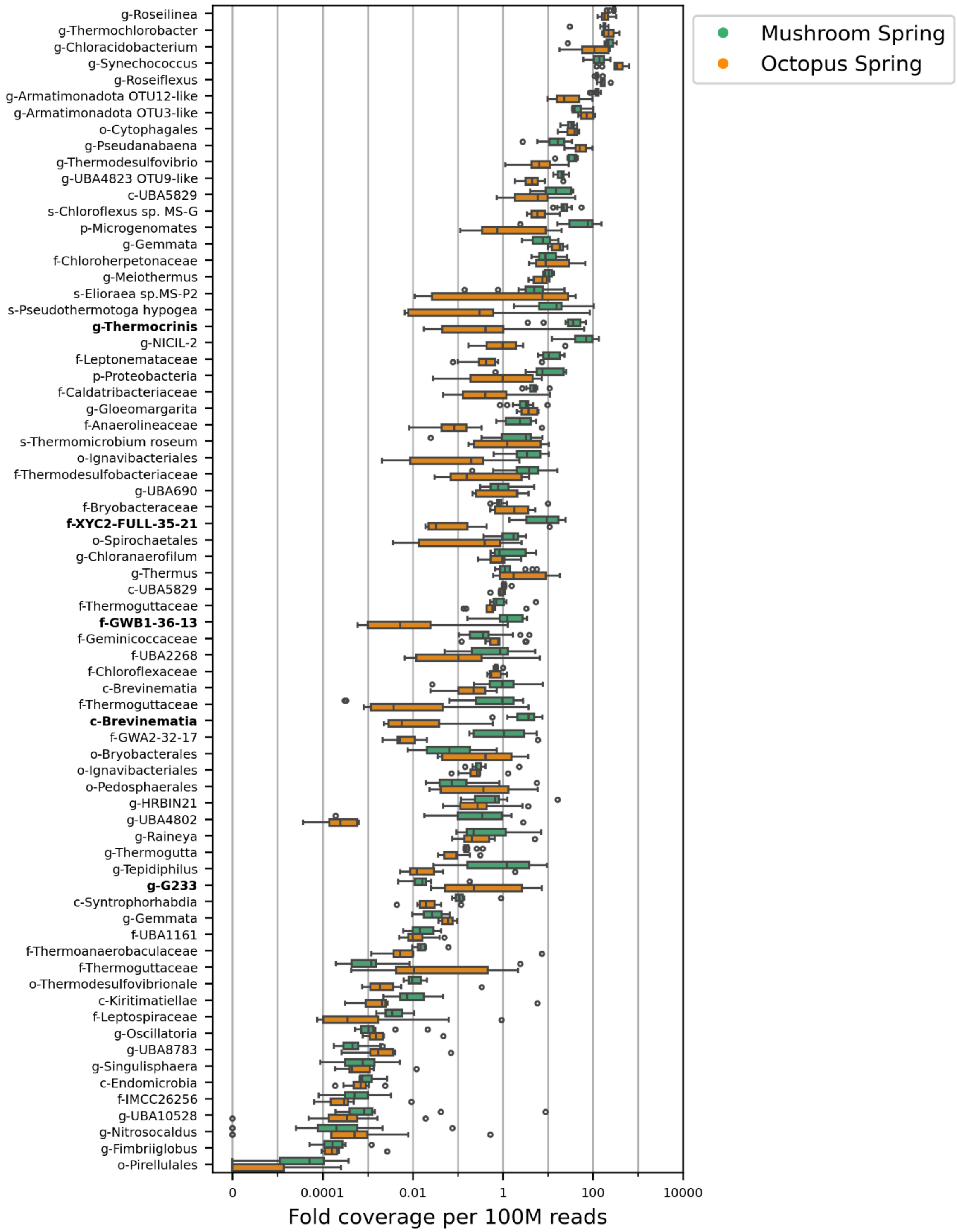


**Supplementary Figure 3: Summary of metagenome relative abundance at 60°C**
The median, upper and lower quartile of the fold coverage of each pan-genome lineage are depicted in the box plot. Whiskers are 1.5 times the interquartile range, and points are those falling outside the whiskers. **Bold** = significantly different relative abundance by the ANCOM test between springs at 60°C.

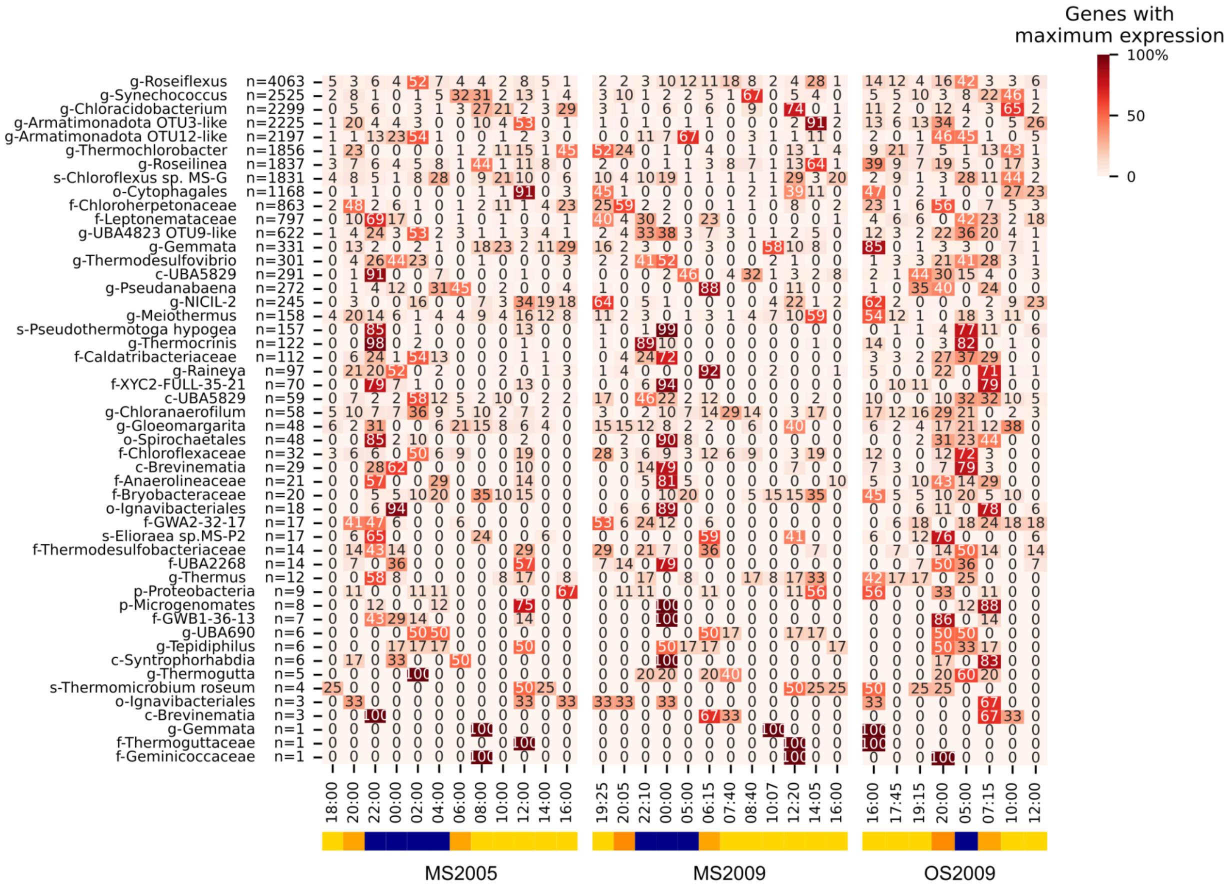


**Supplementary Figure 4: Percent of highly expressed genes per taxon with maximum expression at each timepoint for all three metatranscriptome time series.**
Heatmap of percent of highly expressed gene clusters (mean CPM >1) per taxon that had maximum expression at each time point. n= number of genes analyzed per taxon. Only taxa with at least one highly expressed gene are shown. Bars at the bottom indicate qualitative time of day: yellow: day, orange: dusk or dawn, blue: night (Note that sampling in MS2005 and MS2009 has more time points than OS2009).

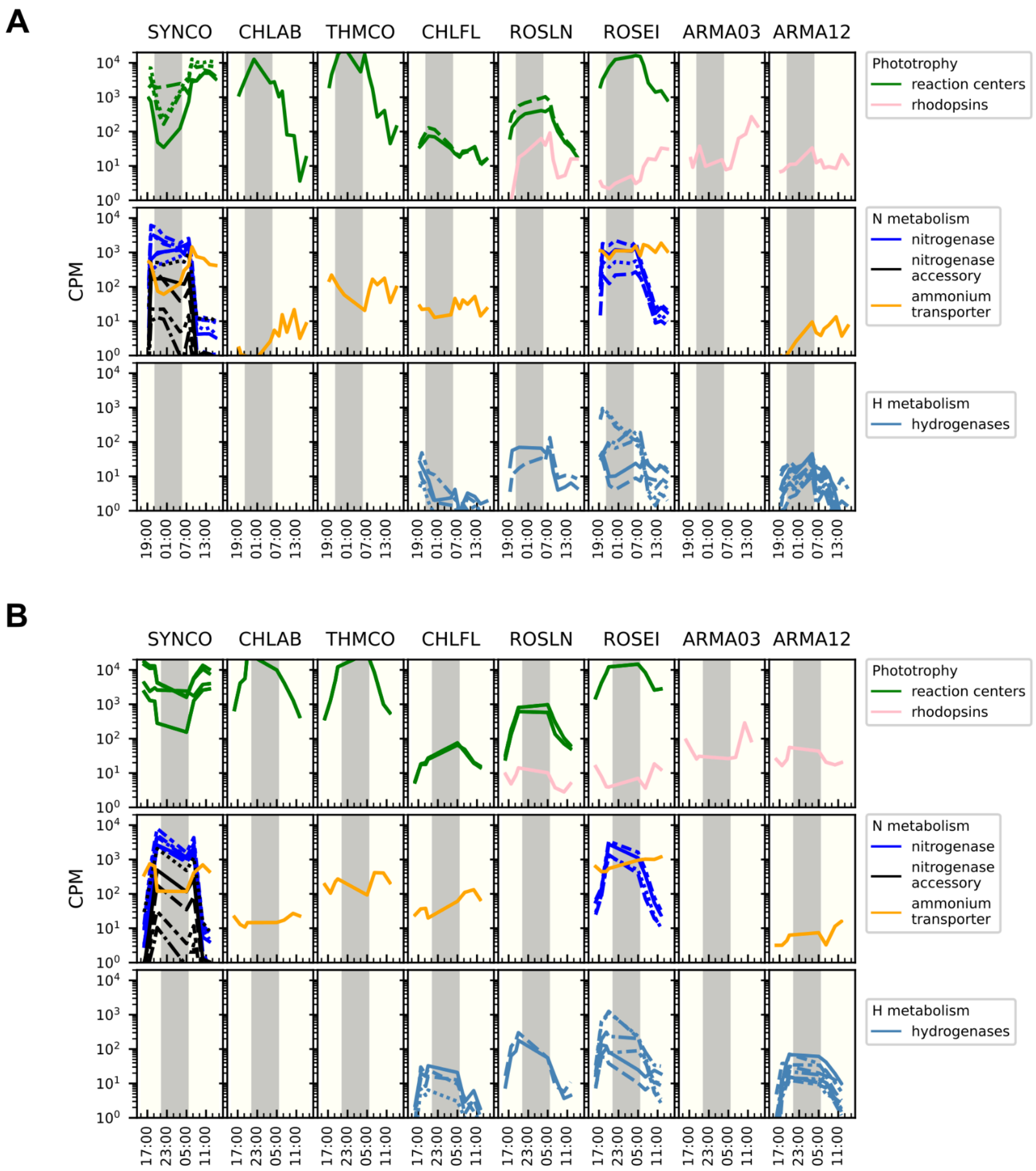


**Supplementary Figure 5: Response of sentinel pathways to the diel cycle in the eight most active taxa in MS2009 and OS2009 datasets**

Gene expression of the same sentinel genes from **Fig. 4** are plotted from the (**A)** MS2009 (**B)** OS2009 metatranscriptome for the eight active taxa to show similarities and differences between the time series. Panels without curves indicate that a gene containing that annotation was not expressed in that taxon. Gray bar: Night period. CPM: counts per million. Underlying data is found in **Supplementary Data 20.**

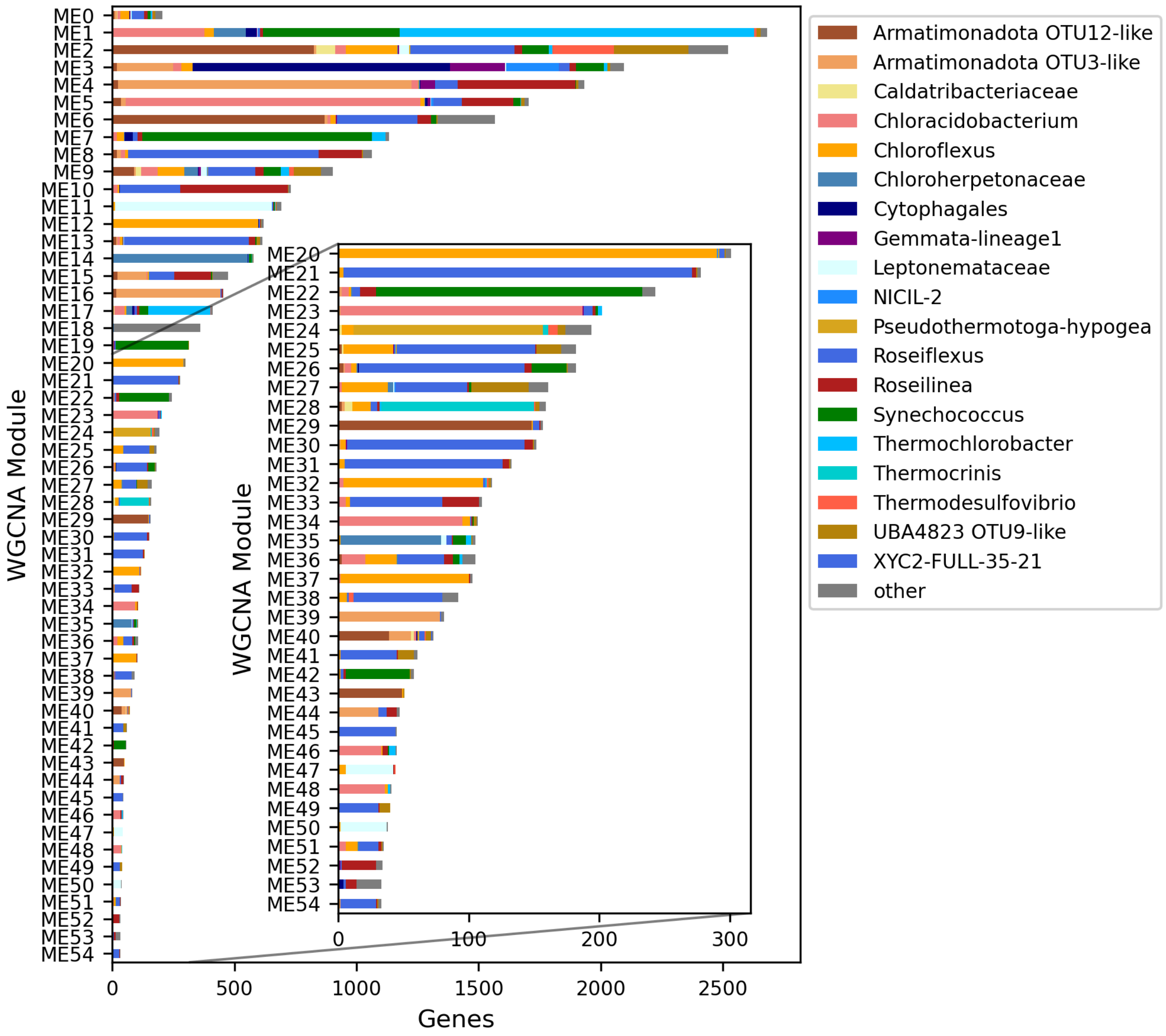


**Supplementary Figure 6: Taxa composition of WGCNA modules**

Taxa composition of genes in consensus WGCNA modules identified in highly expressed genes in all three time series (**Fig. 5**). An inset shows more detail for smaller WGCNA modules.

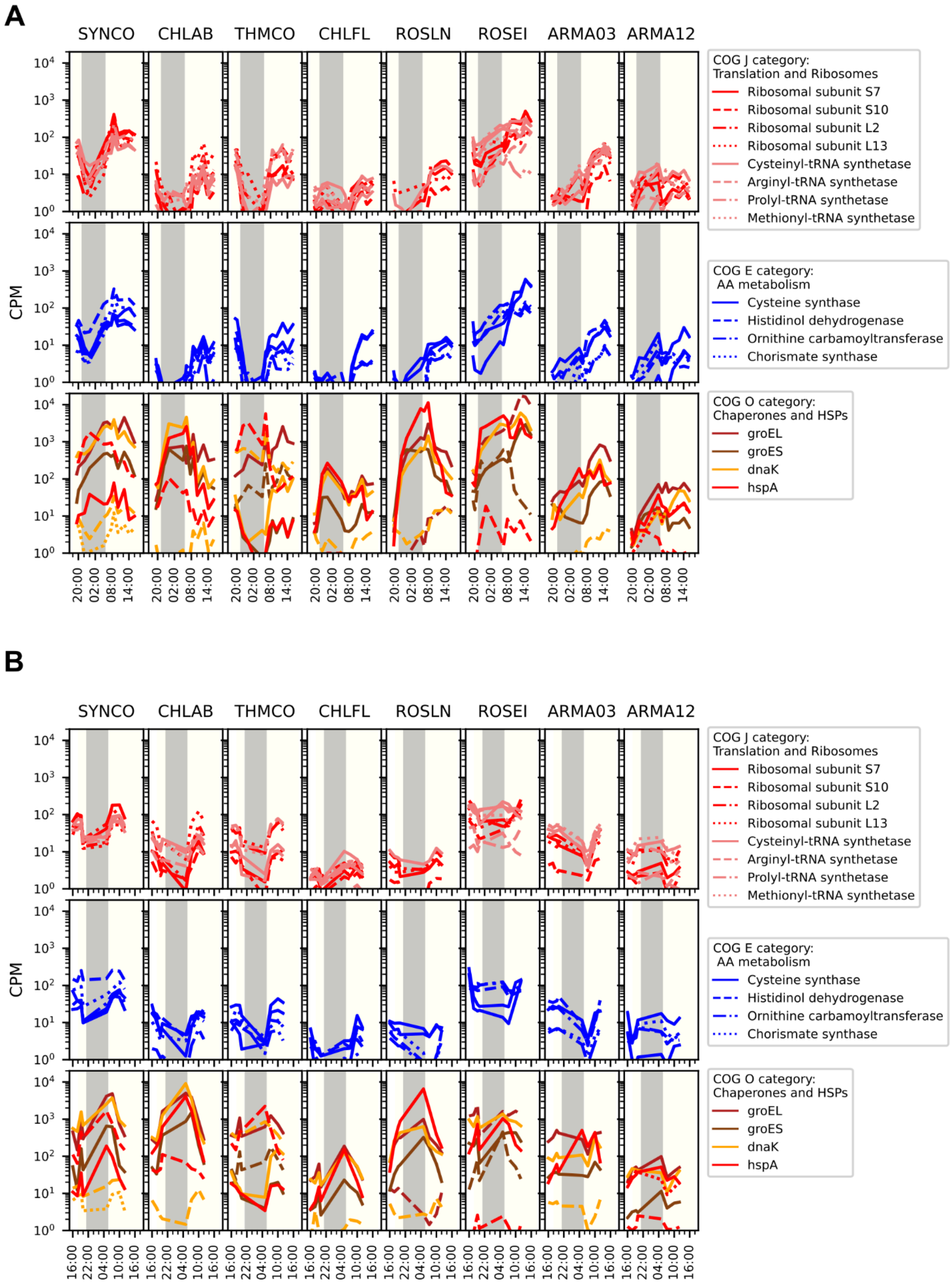


**Supplementary Figure 7: Expression patterns of selected genes from the COG Category overrepresentation analysis in MS2009 and OS2009 datasets**

Gene expression of the same sentinel genes from **Fig. 7** are plotted from the (**a**)MS2009 and (**b**) OS2009 metatranscriptome for the eight active taxa to show similarities and differences between the time series. Each line is a different gene. Top: Category J, Translation and Ribosomes. Middle: Category E, Amino acid (AA) metabolism. Bottom: Category O, Chaperones and heat shock proteins (HSPs). CPM: counts per million. Underlying data is found in **Supplementary Data 21**.

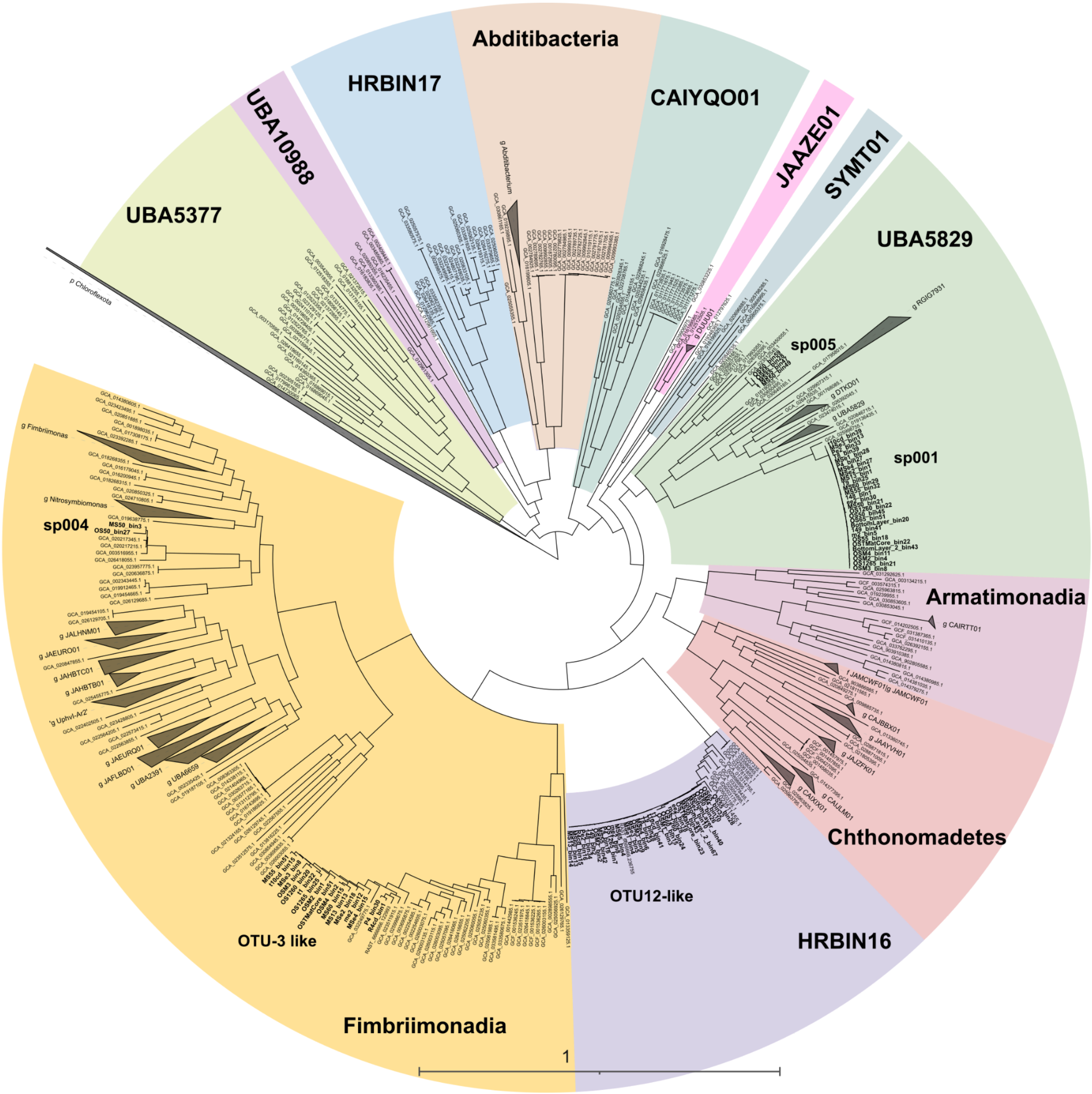


**Supplementary Figure 8: Armatimonadota MAGs in context with available sequences from GTDB and NCBI**

A concatenated single copy protein tree containing GTDB (R207) reference genomes, genomes classified as Armatimonadota on NCBI (downloaded 2023/12/13), the two MAGs from Thiel 2016, 2017, and the Armatimonadota MAGs from this study (bolded text) was created using GTDB-tk. Class assignments by GTDB-tk are indicated by colored backgrounds. Scale bar is amino acid substitutions per site.
